# LEVELS OF EVIDENCE IN ONCOLOGIC-ORTHOPEDIC STUDIES - ACTA ORTOP BRAS (1993-2022)

**DOI:** 10.1590/1413-785220243205e285265

**Published:** 2024-10-28

**Authors:** Alex Guedes, Ângelo Rebouças Fernandes Curvelo Sousa, Marco Aurélio Santos Santana, Aparecida Aguiar Lima Guedes, Ricardo Gehrke Becker, Olavo Pires de Camargo

**Affiliations:** 1. Grupo de Oncologia Ortopédica, Hospital Santa Izabel, Santa Casa de Misericórdia da Bahia, Salvador, BA, Brazil.; 2. Programa de Residência Médica em Ortopedia e Traumatologia, Hospital Santa Izabel, Santa Casa de Misericórdia da Bahia, Salvador, BA, Brazil.; 3. Faculdade de Medicina, Universidade Salvador, Salvador, BA, Brazil.; 4. Departamento de Ortopedia e Trauma, Hospital de Clínicas de Porto Alegre, Porto Alegre, RS, Brazil.; 5. Instituto de Ortopedia e Traumatologia, Hospital das Clinicas, Faculdade de Medicina, Universidade de Sao Paulo(IOT-HC-FMUSP), Sao Paulo, SP, Brazil.

**Keywords:** Evidence-Based Medicine, Epidemiologic Methods, Neoplasms, Orthopedics, Research Design, Periodicals as Topic, Medicina Baseada em Evidências, Métodos Epidemiológicos, Neoplasias, Ortopedia, Projetos de Pesquisa, Publicações Periódicas como Assunto

## Abstract

Objectives: to identify oncological-orthopedic studies published in Acta Ortopédica Brasileira over three decades; to classify them according to the type and level of evidence (LE); to observe the inter-rater agreement in the classification of studies; to analyze the studies retrospectively, according to levels of evidence; and to outline the evolution of the evidence in the study period. Methods: Descriptive analyses were performed with absolute and relative frequencies of studies published between 1993 and 2022. Inter-rater agreement was analyzed by percentage of agreement and Kappa statistic (95%CI). The interpretation of the magnitude of the agreement was performed according to Landis & Koch. The association between classifications and publication period was analyzed using Fisher’s exact test. The analyses were performed using the R program (significance of 5%). Results: 69/1349 papers were selected; there was a significant association between type of study, statistical methodology, and LE with publication period (p < 0.05); inter-rater agreement regarding LE was 92.8%. Conclusions: Oncological-orthopedic studies accounted for 5.1% of all published papers. Regarding the LE, 80% were NE IV and V studies, despite the evolution observed between the first and last decade (decrease in LE V studies and increase in LE II, III and IV). ***Level of Evidence III, Retrospective Comparative Study.***

## RESUMO

Objetivos: identificar estudos oncológico-ortopédicos publicados na *Acta Ortopédica Brasileira* ( *Acta Ortop Bras* ) ao longo de três décadas; classificá-los quanto ao tipo e nível de evidência (NE); observar a concordância interavaliadores na classificação dos estudos; analisar os trabalhos retrospectivamente, de acordo com níveis de evidência; e traçar os perfis evolutivos das evidências no período avaliado. Métodos: Realizou-se análises descritivas com frequências absolutas e relativas dos estudos publicados entre 1993 e 2022. A concordância interavaliadores foi analisada pela porcentagem de concordância e estatística Kappa (IC95%). A interpretação da magnitude da concordância foi realizada de acordo com Landis & Koch. A associação entre classificações e período de publicação foi analisada pelo teste exato de Fisher. As análises foram realizadas no programa R (significância de 5%). Resultados: foram selecionados 69 de um total de 1349 artigos; houve associação significativa entre tipo de estudo, metodologia estatística e NE com período de publicação (p < 0,05); a concordância interavaliadores quanto ao NE foi de 92,8%. Conclusões: Os estudos oncológico-ortopédicos corresponderam a 5,1% de todos os artigos publicados. Quanto ao NE, 80% foram estudos NE IV e V, apesar da evolução observada entre a primeira e a última década (decréscimo de estudos NE V e aumento de NE II, III e IV). ***Nível de Evidência III, Estudo Retrospectivo Comparativo.***

## INTRODUCTION

 Evidence-based medicine (EBM) is an approach that seeks to use the best available scientific evidence to guide medical decisions ^1^ ^-^ ^9^ that are appropriate to patients’ values and preferences. ^5^ Scientific evidence can modify actions, allocate research resources, and influence healthcare decision-makers. ^10^

 The systematic approach to EBM involves, initially, a critical evaluation and stratification of studies into hierarchical levels of evidence. ^11^ ^,^ ^12^ The stratification of evidence is the central element in distinguishing between low- and high-quality studies, which is essential amid the increasing number of studies year after year. ^13^

 Much has been done to disseminate the concepts of EBM that apply to the particular characteristics of orthopedics, along with the critical evaluation of the methodological quality of published studies. ^14^ This is particularly important when considering orthopedic oncology, a subspecialty of orthopedics that deals with neoplasms that affect the musculoskeletal system, characterized by a wide spectrum of rare pathologies, with case records and follow-up that are often insufficient to provide evidence that promotes clinical practice. It has become indispensable to critically analyze the literature for orthopedic oncologists in need of updates, that may be seeking a basis for their conduct in the face of the most diverse pathologies. 

 The journal Acta Ortopédica Brasileira ( *Acta Ortop Bras* ), a publication specialized in Orthopedics and Traumatology with bimonthly periodicity and indexed in PubMed, PubMed Central, Web of Science, SciELO, SCOPUS, Redalyc and LILACS, has achieved great relevance in the Brazilian orthopedic oncology environment since its creation (1993), and is one of the most consulted sources of research in this field. This motivated us to trace an evolutionary line of publications on topics related to orthopedic oncology in this journal. 

 The objectives of this study were: to identify the orthopedic oncology studies published in the journal *Acta Ortop Bras* over three decades (1993-2002, 2003-2012 and 2013-2022); to classify the types of studies and the levels of evidence according to EBM criteria; to observe the inter-rater agreement in the classification of the included studies; to analyze the studies retrospectively, according to their levels of evidence; and to trace an evolutionary profile of the evidence between the three decades in the time series considered. 

## METHODS

 Two researchers independently evaluated all studies published since the first edition of *Acta Ortop Bras* , from 1993 to the year 2022. The studies were compiled from two databases, a promotional CD-ROM ^15^ with the first 15 years of *Acta Ortop Bras* (containing all publications between 1993 and 2007), and the journal’s own website ^16^ (containing all publications between 2000 and 2022). The studies related to orthopedic oncology were selected based on the titles and classified as eligible, potentially eligible, and not eligible. After this initial screening, eligible and potentially eligible studies were screened again, first by reading the abstracts and then in full. A third evaluator resolved any disagreements. 

Descriptive analyses of the data were then performed with absolute and relative frequencies. The inter-rater agreement regarding the level of evidence of the articles was analyzed by the percentage of agreement and the Kappa statistic, with the respective confidence interval (95%CI).

 The interpretation of the magnitude of the inter-rater agreement was performed according to Landis and Koch. ^17^

The associations of the classifications with the period of publication of the article were analyzed using Fisher’s exact test.

 All analyses were performed using the R program, ^18^ with a significance level of 5%. 

## RESULTS

**Figure 1.**
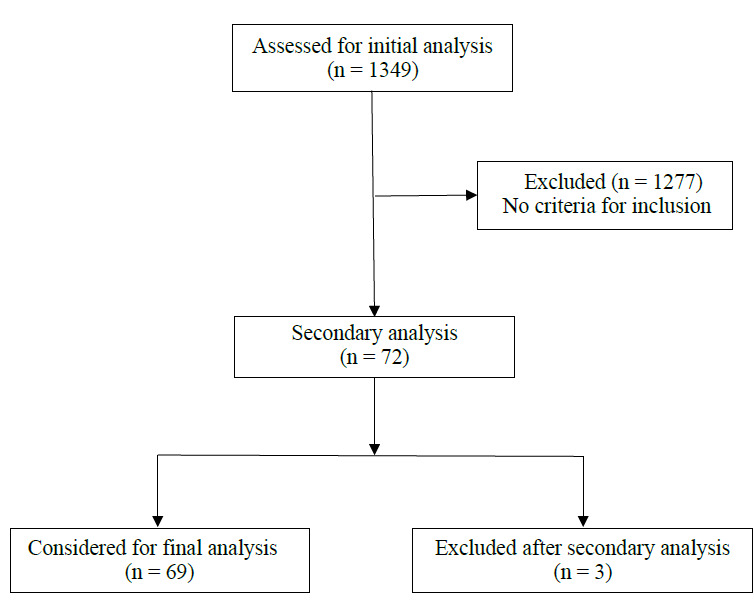
Flow chart.

 Among the 1349 studies published in *Acta Ortop Bras* between 1993 and 2022, we identified 72 eligible studies related to orthopedic oncology. After complete reading, we identified that one of the studies was conducted with rat samples, another with bone samples (femur) and a third evaluated specimens composed of cement cylinders. Thus, 95.8% (n = 69) represented studies involving human beings, constituting the focus of subsequent analyses ( Figure 1 , Table 1 ). 

**Table 1.** Distribution of papers according to characteristics (n = 72).

Features		Period		Total
	1993-2002	2003-2012	2013-2022	
Total Papers	15 (20.8%)	20 (27.8%)	37 (51.4%)	72 (100.0%)
Papers with specimens of animals, bones, or specimens	1 (6.7%)	0 (0.0%)	2 (5.4%)	3 (4.2%)
Papers involving human subjects, assessed for the level of evidence	14 (93.3%)	20 (100.0%)	35 (94.6%)	69 (95.8%)

The analyses that followed considered the division of the studies into publication periods, with the first period referring to papers published between 1993 and 2002, the second period between 2003 and 2012, and the third period between 2013 and 2022.

**Figure 2.**
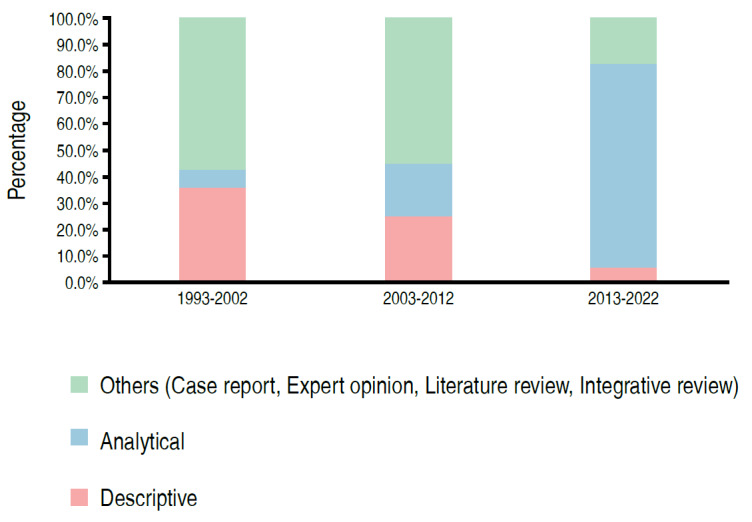
Distribution of papers according to the period and form of data analysis (n = 69).

**Figure 3.**
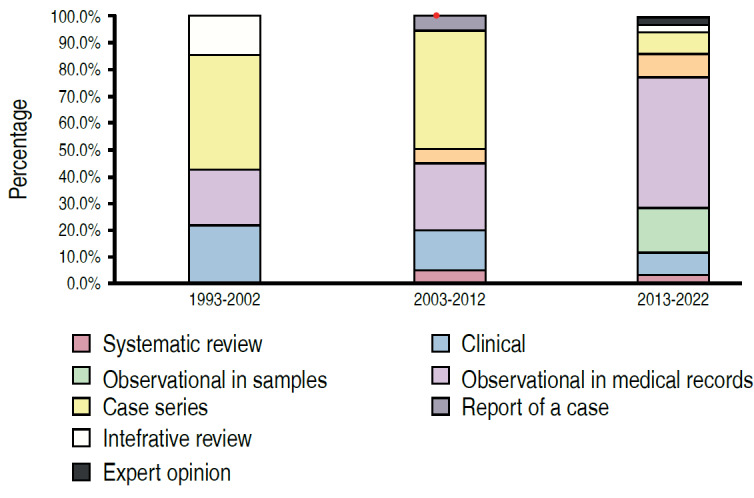
Distribution of papers according to period and type of study (n = 69).

**Figure 4.**
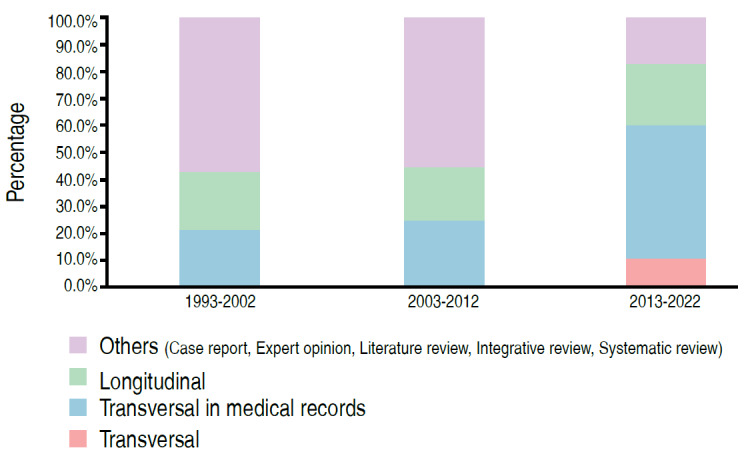
Distribution of papers according to period and observation strategy (n = 69).

**Figure 5.**
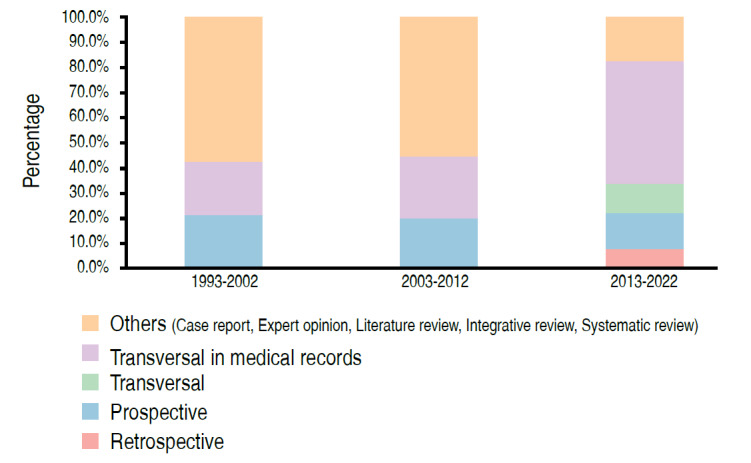
Distribution of papers according to period and temporality (n = 69).

 There was a significant association between the type of study and the period of publication (p < 0.05) ( Table 2 , Figures 2 - 5 ). We can observe that the percentage of papers published with only descriptive studies decreased from 35.7% in the period from 1993 to 2002 to 5.7% in the period from 2013 to 2022. There was also an increase in the percentage of papers with an analytical approach, from 7.1% to 77.1% of the papers published in these periods. There was also a decrease in the percentage of case reports, from 42.9% to 8.6% of the published papers (as of 2011 case reports were no longer accepted at *Acta Ortop Bras* ), with an increase in the number of observational studies in medical records, from 21.4% of the studies in the first period evaluated to 48.6% in the last period. 

**Table 2.** Distribution of papers evaluating samples with human beings according to the type of design used in the study (n = 69).

Feature	Category	Period			Total
		1993-2002	2003-2012	2013-2022	
	Descriptive	5 (35.7%)	5 (25.0%)	2 (5.7%)	12 (17.4%)
	Analytic	1 (7.1%)	4 (20.0%)	27 (77.1%)	32 (46.4%)
	Other (Case report, Expert opinion, Literature review, Integrative review)	8 (57.1%)	11 (55.0%)	6 (17.1%)	25 (36.2%)
p-value		<0.0001			
Type of Study	Systematic review	0 (0.0%)	1 (5.0%)	1 (2.9%)	2 (2.9%)
	Clinical	3 (21.4%)	3 (15.0%)	3 (8.6%)	9 (13.0%)
	Observational in samples	0 (0.0%)	0 (0.0%)	6 (17.1%)	6 (8.7%)
	Observational in medical records	3 (21.4%)	5 (25.0%)	17 (48.6%)	25 (36.2%)
	Case series	0 (0.0%)	1 (5.0%)	3 (8.6%)	4 (5.8%)
	Case report	6 (42.9%)	9 (45.0%)	3 (8.6%)	18 (26.1%)
	Integrative review	2 (14.3%)	0 (0.0%)	1 (2.9%)	3 (4.4%)
	Narrative review	0 (0.0%)	1 (5.0%)	0 (0.0%)	1 (1.4%)
	Expert opinion	0 (0.0%)	0 (0.0%)	1 (2.9%)	1 (1.4%)
p-value		0.0074			
Observation strategy	Cross-sectional	0 (0.0%)	0 (0.0%)	4 (11.4%)	4 (5.8%)
	Cross-sectional in medical records	3 (21.4%)	5 (25.0%)	17 (48.6%)	25 (36.2%)
	Longitudinal	3 (21.4%)	4 (20.0%)	8 (22.9%)	15 (21.7%)
	Other (Case report, Expert Opinion, Review Literature Review, Integrative Review, Systematic review)	8 (57.1%)	11 (55.0%)	6 (17.1%)	25 (36.2%)
p-value		0.0282			
Temporality	Retrospective	0 (0.0%)	0 (0.0%)	3 (8.6%)	3 (4.3%)
	Prospective	3 (21.4%)	4 (20.0%)	5 (14.3%)	12 (17.4%)
	Cross-sectional	0 (0.0%)	0 (0.0%)	4 (11.4%)	4 (5.8%)
	Cross-sectional in medical records	3 (21.4%)	5 (25.0%)	17 (48.6%)	25 (36.2%)
	Other (Case report, Literature Review, Integrative Review, Systematic Review)	8 (57.1%)	11 (55.0%)	6 (17.1%)	25 (36.2%)
p-value		0.0255			

**Table 3.** Distribution of papers evaluating samples with human beings as a function of statistical analysis (n=69).

Sample Feature	Category		Period		Total
		1993-2002	2003-2012	2013-2022	
Sample calculation presented	No	6 (42.9%)	9 (45.0%)	24 (68.6%)	39 (56.5%)
	No, but sample size is a limitation of the study	0 (0.0%)	1 (2.9%)	1 (1.4%)	4 (5.8%)
	Yes	0 (0.0%)	0 (0.0%)	1 (2.9%)	1 (1.4%)
	Not applicable	8 (57.1%)	11 (55.0%)	6 (17.1%)	25 (36.2%)
p-value		0.0147			
Applied methodology statistics for analyze the data		5 (35.7%)	5 (25.0%)	2 (5.7%)	12 (17.4%)
		1 (7.1%)	4 (20.0%)	27 (77.1%)	32 (46.4%)
	Not applicable	8 (57.1%)	11 (55.0%)	6 (17.1%)	25 (36.2%)
p-value		<0.0001			
It presented the power of the test, size of effect or confidence interval	No	6 (42.9%)	8 (40.0%)	22 (62.9%)	36 (52.2%)
	Confidence interval	0 (0.0%)	1 (5.0%)	6 (17.1%)	7 (10.1%)
	Effect Size	0 (0.0%)	0 (0.0%)	0 (0.0%)	1 (1.4%)
	Test Power	0 (0.0%)	0 (0.0%)	1 (2.9%)	25 (36.2%)
	Not applicable	8 (57.1%)	11 (55.0%)	6 (17.1%)	25 (36.2%)
p-value		0.0193			

**Figure 6.**
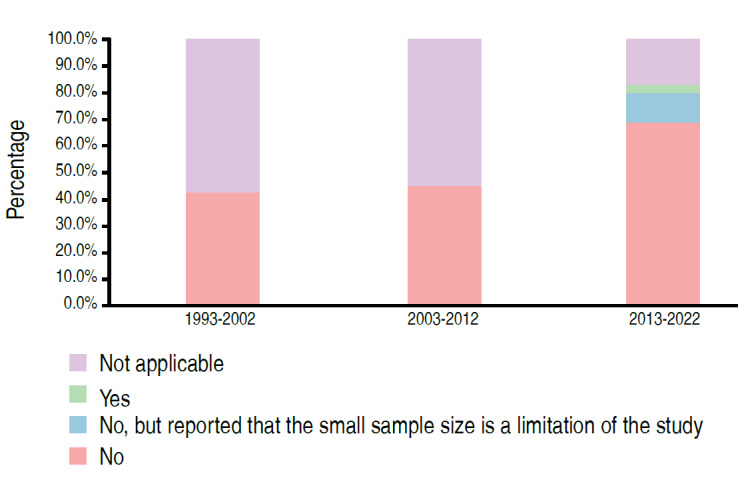
Distribution of papers according to period and sample calculation presentation (n=69).

**Figure 7.**
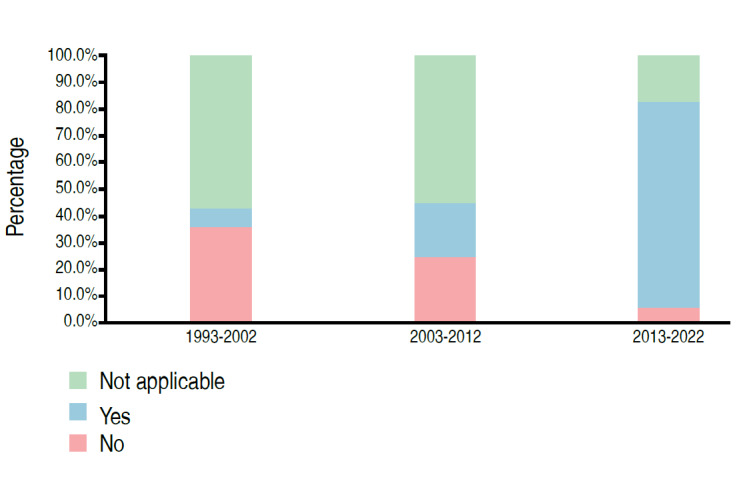
Distribution of papers according to period and application of statistical methodology to analyze data (n=69).

**Figure 8.**
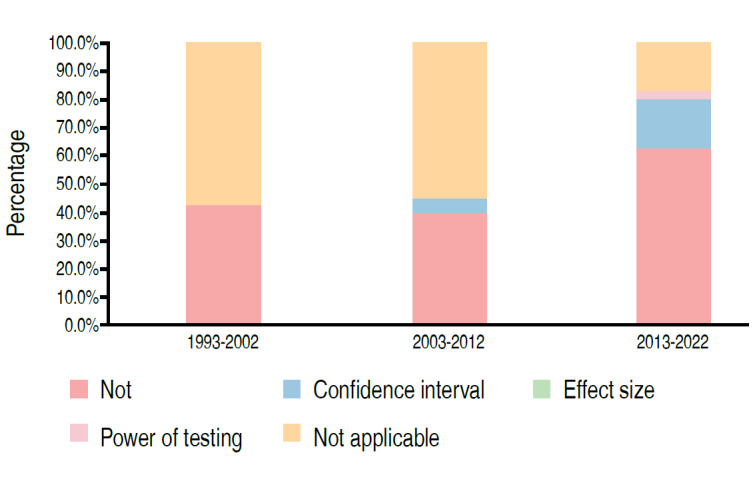
Distribution of papers according to the period and presentation of test power, effect size or confidence interval (n=69).

**Figure 9.**
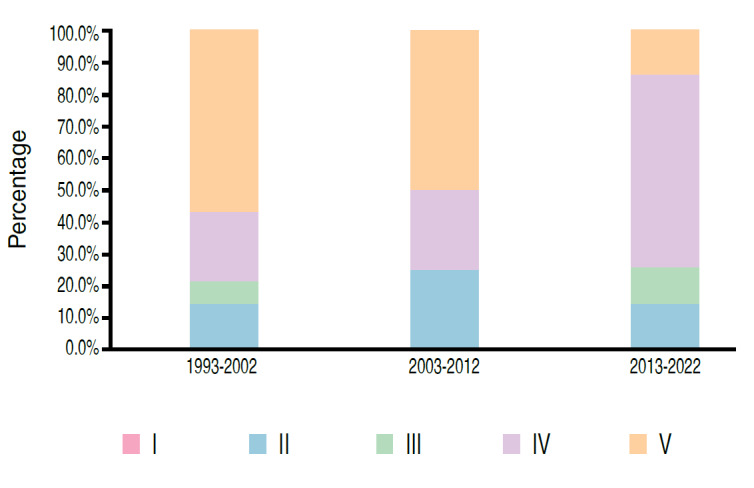
Distribution of papers according to level of evidence (n = 69).

 The inter-rater agreement regarding the level of evidence of the published papers, according to the table provided by the journal, was 92.8%, classified as almost perfect agreement according to Landis and Koch ^13^ (Kappa = 0.89) ( Table 4 ). 

**Table 4.** Results of the inter-rater reproducibility analysis for the level of evidence of the papers (n = 69).

Statistics	Value
Agreement	92.8%
Weighted Kappa (CI95%)	0.89 (0.80-0.99)

 CI: Confidence interval. Classification of reproducibility according to Landis and Koch ^4^ : Almost perfect agreement. 

 The inter-rater cases of disagreement were presented to a third evaluator and the final level of evidence is presented in Table 5 and Figure 9 . A significant association was observed between the level of evidence of the study and the period of paper publication (p < 0.05). There was a decrease in the percentage of papers with evidence level V, from 57.1% in the period from 1993 to 2002 to 14.3% in the period from 2013 to 2022. On the other hand, the percentage of papers with evidence level IV increased from 21.4% to 60.0%. There was also a slight increase in the percentage of papers with level III evidence, from 7.1% to 11.4% of published papers. 

**Table 5.** Distribution of papers evaluating samples with human beings according to the level of evidence (n = 69).

		Period		
Level of evidence				Total
	1993-2002	2003-2012	2013-2022	
I	0 (0.0%)	0 (0.0%)	0 (0.0%)	0 (0.0%)
II	2 (14.3%)	5 (25.0%)	5 (14.3%)	12 (17.4%)
III	1 (7.1%)	0 (0.0%)	4 (11.4%)	5 (7.2%)
IV	3 (21.4%)	5 (25.0%)	21 (60.0%)	29 (42.0%)
V	8 (57.1%)	10 (50.0%)	5 (14.3%)	23 (33.3%)
p-value	0.0056			

## DISCUSSION

 The number of orthopedic oncology publications in *Acta Ortop Bras* over the study period was restricted; of the total 1349 papers published in thirty years, only 5.1% referred to this orthopedic subspecialty, which indicates the need to stimulate further scientific research in the national reference centers of this subspecialty. 

 The most frequent study designs were case reports, case-control studies, retrospective-comparative studies, systematic reviews of level III studies, and expert opinions, representing approximately 80% of all papers evaluated. Orthopedic publications seem to follow this trend of low level of methodological evidence, as pointed out by Moraes et al. ^10^ in a study on the hierarchy of evidence in hand surgery in Brazilian orthopedic journals and by Kiter et al., ^1^ in the analysis of publications in nine high-impact international orthopedic journals. Orthopedists have been criticized for publishing few studies with a high methodological level; however, since not all questions can be studied with these characteristics, the relative preponderance of lower-level studies may not accurately describe the frequency with which orthopedic researchers use inappropriate means and, in turn, may not accurately represent the quality of the literature on orthopedics. ^19^ The current state of Brazilian research in orthopedic oncology cannot be judged by the findings of our study, since relevant research of high methodological quality is usually published in journals with greater visibility and academic impact. 

In parallel with the above, a significant association was identified between the type of study and the period of publication, since the percentage of papers published only with descriptive studies decreased (37.5% in 1993-2002 to 5.7% in 2013-2022), while the percentage of studies with an analytical approach increased significantly (7.1% to 77.1% in the same period). This was in addition to the perception of an increase in the use of statistical methodology to analyze and validate study data: only 7.1% of the studies used statistics in 1993-2002, while 77.1% used them in 2013-2022. This demonstrates the authors’ concern with the improvement in the methodology of the studies over time.

 We also observed an improvement in the quality of the predominant studies over the decades, since there was a significant drop in the percentage of papers with levels of evidence V (57.1% in 1992-2003 to 14.3% in 2013-2022) as well as a significant increase in the observance of papers with level of evidence IV (21.4% in 1992-2003 to 60%% in 2013-2022) and a discrete increase in the number of papers with level of evidence IV (21.4% in 1992-2003 to 60% in 2013-2022) . There was an evolution in relation to papers with levels of evidence II and III (21.4% in 1992-2003 to 24.6% in 2013-2022). This chronological change, directed to research designs of a higher methodological level, has been identified in similar studies based on historical series of orthopedic journals. ^12^ ^,^ ^20^ Finally, we observed that the inter-rater agreement was classified as almost perfect, conferring good reproducibility to the method of classification of evidence used by the journal, which makes it a viable instrument for the evaluation of studies. 

## CONCLUSIONS

 The orthopedic oncology studies published in *Acta Ortop Bras* during the study period showed a low prevalence (5%) considering the number of studies published on other subspecialties. The level of evidence (LE) of these studies still showed, after three decades, a predominance of studies classified as LE IV and V, despite a significant improvement observed between the first and last decade regarding the decrease in LE V studies and an increase in LE II, III and IV studies; which leads us to believe that high-quality evidence related to orthopedic oncology is still poorly available. This scenario puts researchers in the position to make an effort to produce more randomized clinical trials and meta-analyses for the subspecialty. The inter-rater agreement regarding the level of evidence of the published papers was 92.8%, classified as almost perfect. 
